# Positive End-Expiratory Pressure may alter breathing cardiovascular variability and baroreflex gain in mechanically ventilated patients

**DOI:** 10.1186/1465-9921-11-38

**Published:** 2010-04-19

**Authors:** Andry Van de Louw, Claire Médigue, Yves Papelier, François Cottin

**Affiliations:** 1 Unité de Biologie Intégrative des Adaptations à l'Exercice (INSERM 902/EA 3872, Genopole), ZAC du Bras de Fer, 3 bis impasse Christophe Colomb, 91000 Evry, France; 2 Intensive Care Unit, Centre Hospitalier Sud-Francilien, Quartier du Canal, Courcouronnes, 91014 Evry, France; 3 Institut National de Recherche en Informatique et en Automatique, Domaine de Voluceau, Rocquencourt, 78153 Le Chesnay, France; 4 EA 3544, EFM, Hôpital Antoine Béclère, 92141 Clamart, France

## Abstract

**Background:**

Baroreflex allows to reduce sudden rises or falls of arterial pressure through parallel RR interval fluctuations induced by autonomic nervous system. During spontaneous breathing, the application of positive end-expiratory pressure (PEEP) may affect the autonomic nervous system, as suggested by changes in baroreflex efficiency and RR variability. During mechanical ventilation, some patients have stable cardiorespiratory phase difference and high-frequency amplitude of RR variability (HF-RR amplitude) over time and others do not. Our first hypothesis was that a steady pattern could be associated with reduced baroreflex sensitivity and HF-RR amplitude, reflecting a blunted autonomic nervous function. Our second hypothesis was that PEEP, widely used in critical care patients, could affect their autonomic function, promoting both steady pattern and reduced baroreflex sensitivity.

**Methods:**

We tested the effect of increasing PEEP from 5 to 10 cm H2O on the breathing variability of arterial pressure and RR intervals, and on the baroreflex. Invasive arterial pressure, ECG and ventilatory flow were recorded in 23 mechanically ventilated patients during 15 minutes for both PEEP levels. HF amplitude of RR and systolic blood pressure (SBP) time series and HF phase differences between RR, SBP and ventilatory signals were continuously computed by complex demodulation. Cross-spectral analysis was used to assess the coherence and gain functions between RR and SBP, yielding baroreflex-sensitivity indices.

**Results:**

At PEEP 10, the 12 patients with a stable pattern had lower baroreflex gain and HF-RR amplitude of variability than the 11 other patients. Increasing PEEP was generally associated with a decreased baroreflex gain and a greater stability of HF-RR amplitude and cardiorespiratory phase difference. Four patients who exhibited a variable pattern at PEEP 5 became stable at PEEP 10. At PEEP 10, a stable pattern was associated with higher organ failure score and catecholamine dosage.

**Conclusions:**

During mechanical ventilation, stable HF-RR amplitude and cardiorespiratory phase difference over time reflect a blunted autonomic nervous function which might worsen as PEEP increases.

## Background

Autonomic nervous system plays a crucial role in the maintenance of circulatory homeostasis. Several studies have documented the worse prognosis associated with autonomic dysfunction in sepsis [1], trauma [2] or multiple organ failure [3]. In order to assess the autonomic nervous function, these studies mainly investigated heart rate variability or baroreflex sensitivity. Baroreflex allows to compensate for sudden changes in arterial pressure, through baroreceptors activation and subsequent stimulation or inhibition of autonomic pathways located in the brainstem, which induce parallel changes in RR intervals [4].

Many factors may interfere with the autonomic nervous system in critical care patients, like significant comorbidities (diabetes mellitus [5], ischemic heart disease [6]), sedative [7] or vasoactive [8] drugs. During spontaneous breathing, the use of positive end-expiratory pressure (PEEP) may also affect the autonomic nervous system function, with conflicting results. PEEP increased the high frequency heart rate variability in congestive heart failure [9] and in patients with obstructive sleep apnea, with [10] or without [11] associated heart failure. The baroreflex sensitivity was acutely improved by PEEP in obstructive sleep apnea [12], but pressure levels > 10 cm H2O were associated with a decreased baroreflex sensitivity in healthy subjects [13]. In critical care patients, PEEP is also widely used during non-invasive or invasive mechanical ventilation. PEEP was recommended in the management of critical care patients with acute lung injury [14], in order to reduce the proportion of nonaerated lung and to improve arterial oxygenation. Nevertheless, its effect on autonomic nervous function has been poorly explored in mechanically ventilated patients.

During mechanical ventilation, we have recently observed that high-frequency amplitude of RR variability (HF-RR amplitude) and cardiorespiratory phase difference were very steady over time in some patients, and extremely variable in others [15]. Conversely, for systolic blood pressure (SBP), HF-SBP amplitude and phase were stable in all patients, with an inversion of SBP phase during the ventilator cycle compared to spontaneous breathing with negative inspiratory pressure. These findings suggested that the direct mechanical effect of intrathoracic positive pressure on SBP was the main determinant of SBP variability, whereas RR variability depended on both mechanical and autonomic nervous factors. By analogy to the arterial pressure, a stable pattern of HF-RR amplitude and cardiorespiratory phase difference could reflect a marked mechanical effect overwhelming a blunted autonomic nervous system function. Moreover, complexity of RR variability is controlled by the autonomic nervous system [16], and many pathological states are characterized by loss of autonomic tone as well as most markers of heart rate complexity [17]. Thus, the stable pattern that we have observed in some mechanically ventilated patients could be related to an autonomic dysfunction.

The first hypothesis of the present study was that a steady pattern of RR variability, in amplitude (HF-RR amplitude) and phase (cardiorespiratory phase difference) could be associated with reduced baroreflex sensitivity and mean HF-RR amplitude, reflecting a blunted autonomic nervous function. As PEEP has been shown to affect the autonomic nervous function in other settings, our second hypothesis was that increasing PEEP could further worsen the autonomic nervous function, promoting both steady pattern and reduced baroreflex sensitivity.

## Methods

This prospective observational study was conducted between November 2007 and September 2008 in the 16-bed medical-surgical intensive care unit (ICU) of the Sud-Francilien General Hospital (Evry, France). The study protocol was approved by the ethics committee of the Francophone Society for Critical Care (CE-SRLF 08-273), who waived the need for written informed consent. Nevertheless, an information letter was given to the patients and/or close relatives, indicating the possibility for the patients to refuse the use of their data.

### Inclusion criteria

According to the definition of the American-European Consensus Conference on acute respiratory distress syndrome [18], patients with acute lung injury (ALI) and for whom an increase in PEEP was prescribed by the attending physician were included. Increasing PEEP is recommended in the treatment of hypoxaemia [14] and is routinely prescribed in our ICU for ALI patients. ALI criteria were acute hypoxemia with a ratio of the partial pressure of arterial oxygen over the fraction of inspired oxygen (PaO_2_/FiO_2_) no greater than 300 mm Hg, bilateral infiltrates consistent with pulmonary oedema on a frontal chest radiograph, and either no clinical evidence of left atrial hypertension or (if measured) a pulmonary-artery wedge pressure no greater than 18 mm Hg. All the study patients received the same dosages of sedative drugs (midazolam and fentanyl) to tolerate mechanical ventilation. In addition, all were equipped with a radial or femoral artery catheter in order to continuously monitor arterial pressure.

### Exclusion criteria

Patients who had pre-existing or new-onset cardiac arrhythmias, treatment with anti-arrhythmic drugs, incomplete adaptation to the ventilator, or no arterial catheter were not included.

### Protocol

The patients were kept in a semi-recumbent position and left undisturbed, with no changes in ventilator parameters or medication during data collection. Ventilator settings were as follows: volume assist-control mode; tidal volume (Vt), 6 ml/kg ideal body weight; breathing rate, 20 cycles/minute; inspiratory/expiratory ratio, 1/2. FiO_2 _was adjusted to maintain transcutaneous oxygen saturation in blood ≥ 94%. All patients were sedated with midazolam and fentanyl in dosages that were titrated to achieve an adequate adaptation to the ventilator settings. Age, gender, aetiology of ALI, SAPS II severity-of-illness score [19], duration of mechanical ventilation and ICU mortality were collected. At PEEP 10, SOFA (organ failure assessment score) [20], vasoactive drugs dosage, FiO_2_, PaO2/FiO_2 _ratio and PaCO2 were also recorded.

#### Signal acquisition

We performed two signal acquisitions for each patient. The first one was carried out at a PEEP of 5 cm H2O and, after a stabilization period of at least 30 minutes, the second one was carried out at a PEEP of 10 cm H2O. These two levels of PEEP were selected in order to provide a range (5-10 cm H2O) large enough to evidence significant differences and to limit the possible deleterious effects of higher PEEP levels [21]. For each signal acquisition, one-lead electrocardiogram (ECG), arterial pressure, and respiratory flow signals were recorded over a 15-min period using a Biopac 100 system (Biopac systems, Goleta, CA, USA). The respiratory flow was measured with a Hans-Rudolph pneumotachograph (Hans Rudolph Inc, Shawnee, KS, USA) connected to a differential pressure transducer (Validyne MP-45; Validyne, Northridge, CA, USA) and an electronic flow integrator (Validyne MC 1-3; Validyne, Northridge, CA, USA). All data were sampled at 1000 Hz and stored on a hard-disk. The data were acquired for all the patients at the same time of the day to ensure comparable circadian influence.

#### Signal analysis

##### Raw data processing

Signal processing was performed using the Scicos-Scilab and Matlab environments at the French National Institute for Research in Computer Science and Control (INRIA -Sisyphe team). For each 15-min recording, the first and last 100 seconds were removed to avoid border effects of signal processing methods. As very few extra-systolic beats or artifact periods were observed, they were not corrected but discarded from the analysis. Thus, 700-second artifact-free periods were available from most of the patients. RR and SBP time series were extracted from ECG and BP raw signals: ECG and BP were multiplied by a parabolic signal, adapted to the QRS/systolic pressure width, along successive windowing epochs. This parabolic fitting enhances their maximal values and minimizes their lower values, improving the detection of the R peak from the ECG and of the systolic value from the BP signal [22]. Vt was computed with Chart5 soft (Chart5, v5.5, ADInstruments, AUS) by integrating the respiratory flow signal after calibration, as previously described [23]. All series (RR, SBP and Vt) were resampled at 4 Hz, by the interpolation of a third order spline function to obtain equidistant data. The series were then analyzed using the following time and frequency methods.

##### Assessing the phase difference between cardiovascular and respiratory signals and the instantaneous breathing amplitude of RR and SBP series

*a) Complex DeModulation (CDM) parameters*

The CDM, a time-local version of harmonic analysis, has been used to measure cardiovascular and respiratory interactions [24-27]. CDM provides an instantaneous and continuous assessment of the amplitude (HF-CDM), frequency, and phase of RR and SBP variabilities with breathing. To reduce noise and to obtain a monocomponent signal, cardiovascular series were first filtered through a narrow band-pass filter centered on the breathing frequency (0.30 - 0.36 Hz). This narrow band differed from the conventional HF band (0.15-0.4 Hz) defined by the Task Force [28]: in healthy individuals breathing spontaneously, breathing frequency can change and the HF range was defined from 0.15 to 0.4 Hz to ensure that the breathing cardiovascular variability peak was contained in the HF range. In our study, tidal volume was delivered by the ventilator at a strictly constant frequency (0.33 Hz). This allows us to determine a narrow band around this central frequency, which contains nearly all the respiratory oscillations and only them, avoiding noise or spectral activity different from them, and providing more accurate data than the classical range [27]. The instantaneous delay between cardiovascular and respiratory phases was assessed based on the actual modulating breathing frequency [26,27].

*b) Choice of a robust criterion for the classification into two groups, stable versus unstable*

As previously defined [15], the classification was first based on a qualitative visual estimation of two patterns of stability for HF-RR amplitude and cardiorespiratory phase difference over time, and was secondarily confirmed by a quantitative estimation.

Stability could be estimated by standard-deviation (SD) of parameters and also by remarkable values such as: amplitude of the maximum drift of cardiorespiratory phase difference, percentage of time spent below a threshold for HF-RR amplitude.

All these parameters being strongly correlated (phase is arctan of amplitude, mean r = 0.9), a single criterion has been chosen, according to HF-RR amplitude rather than cardiorespiratory phase difference. Indeed, diminutions/disappearances of HF-RR amplitude were always associated with cardiorespiratory phase changes whereas the inverse proposition was not always verified [27]. The percentage of time spent below a threshold was preferred to SD because it gave priority to diminutions/disappearances of respiratory oscillations. The threshold value, fixed to 50% of the mean HF-RR amplitude for the whole period, seemed to be a good compromise, comforted by strongly significant differences of all parameters between the two groups.

Therefore, patients with HF-RR amplitude never decreasing below this threshold were defined as stable, whereas patients who exhibited falls of HR-RR amplitude below the threshold were defined as unstable.

##### Assessing baroreflex sensitivity (BRS)

The smoothed power spectral density (SPSD) was used to quantify local SBP and RR respiratory variabilities, which served to assess baroreflex sensitivity. SPSD has been already described [26,27]. SPSD was applied to successive 64-point Hanning windows (16 sec, therefore containing 5 respiratory oscillations) for each cardiovascular series.

Spectral power was computed in the same high-frequency (HF) band as for CDM analysis, by integrating the power spectral density in the RR and SBP spectra.

A cross spectral analysis was applied to RR and SBP spectra to compute the Coherence and Transfer functions [26,27]. The spectral BRS is supported by the hypothesis of a linear relation between the input (BP) and output (RR) of the model. The degree of linearity between the two signals is estimated by the value of the Coherence function. It was accepted that RR and SBP spectra had a reliable linear relationship when the coherence index was higher than 0.5 [29,30].

The averaged spectral gain in the HF band was the modulus of the transfer function between the RR and SBP spectra [31,32].

### Statistical analysis

All results were reported as means ± SEM when normality has been checked, as median (25-75 percentile) values otherwise. The normality of the data was checked with a Kolmogorov-Smirnov test. To compare PEEP 5 versus PEEP 10 in all subjects, paired tests were applied: the parametric t-test (one way repeated Anova measures) in case of normality, the non parametric Wilcoxon test (repeated measures on ranks) otherwise. To compare the two groups at PEEP 10, unpaired tests were applied: the parametric t-test in case of normality, the non parametric Mann-Whitney rank sum test otherwise.

## Results

### Patients

We included 23 patients (15 men and 8 women). ALI was due to aspiration pneumonia (10 patients), community acquired pneumonia (3 patients), peritonitis (5 patients), severe acute pancreatitis (2 patients), hemorrhagic shock (2 patients) and necrotizing cellulitis (1 patient). 16 patients received vasoactive drugs and 12 died in the ICU. Characteristics of the patients were summarized in Table 1. Among the data recorded, SOFA (Sequential Organ Failure Assessment) score and vasoactive drug dosage were significantly higher in the stable group compared with the unstable one. The other parameters did not differ between the two groups.

**Table 1:** Characteristics of the patients and comparison between the two groups (unstable *vs *stable) at PEEP 10.

Parameters	All patients (n = 23)	Unstable group (n = 11)	Stable group (n = 12)	*p *(unstable *vs *stable group)
Age (years)	55 (49-61)	53 (49-60)	56 (49-61)	0.90
SAPS II	61 (49-75)	58 (46-61)	71 (53-79)	0.13
Duration of mechanical ventilation (days)	12 (7-15)	12.0 (6.5-21.8)	12.5 (9.5-15.0)	0.74
PaO_2_/FiO_2_	210 (150-293)	242 (154-375)	175 (150-266)	0.48
FiO_2 _(%)	50 (40-60)	45 (36-59)	50 (40-70)	0.54
PaCO_2 _(mmHg)	39 (37-45)	40 (37-44)	38 (36-46)	0.76
SOFA score	9.0 (6.3-11.8)	7.0 (5.3-10.0)	10.5 (7.5-14.5)	0.04
Vasoactive drugs dosage (μg.kg^-1^.min^-1^)	0.25 (0.00-0.35)	0.13 (0.00-0.25)	0.32 (0.22-0.78)	0.03

All results were reported as median (25-75 percentile) values. PaO_2_/FiO_2_, FiO_2_, PaCO_2_, SOFA score and vasoactive drugs dosage were collected at PEEP 10. SOFA score and vasoactive drugs dosage were significantly higher in the stable group.

### Autonomic nervous differences between stable and unstable groups at PEEP 10

#### Baroreflex sensitivity (Table 2 and Figure 1)

**Figure 1:**
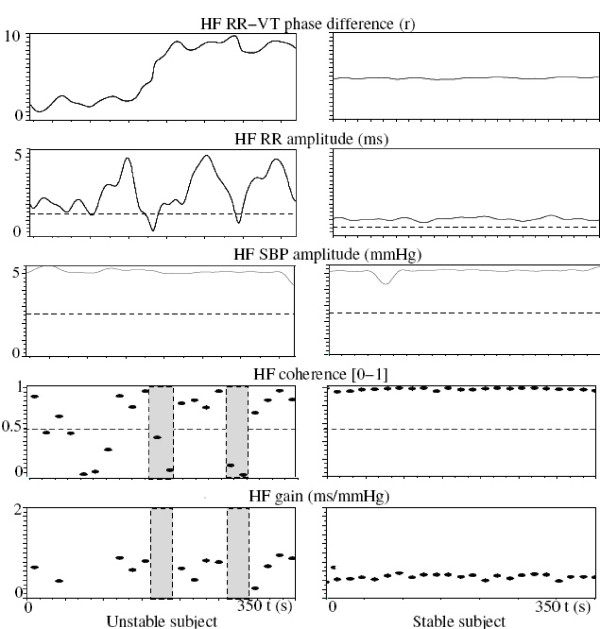
**Comparison between an unstable and a stable subject at PEEP 10, through significant spectral (summarized in Table 2) and CDM (summarized in Table 3) parameters**. At the top, solid lines represented the CDM instantaneous parameters (HF-RR phase and amplitude). At the bottom, each dot represented an averaged value over 16 seconds, from the spectral gain method. The CDM method revealed a greater variability in HF-RR phase and amplitude in the unstable group, with great phase drifts and time spent below the amplitude threshold (dashed line). The spectral gain method revealed a higher gain in the unstable group, due to a greater HF-RR spectral density (close to the HF-RR CDM amplitude). The grey bands represented Fast Fourier Transform epochs with no efficient gain, corresponding to losses of CDM HF-RR amplitude, explaining the great correlation between the two following parameters: the percentage of time (in seconds) spent under an amplitude threshold provided by the CDM and the percentage of time (in epochs of 16 seconds) spent with a coherence value below 0.5.

**Table 2:** Comparison of the spectral gain parameters between the two groups of patients (unstable *vs *stable) at PEEP 10.

Spectral components	Unstable group (n = 11)	Stable group (n = 12)	*p *value
HF-RR spectral density (ms^2^)	6328 (2277-29222)	722 (457-1798)	0.003
HF-SBP spectral density (mmHg^2^)	1048 (496-4279)	1963 (851-3613)	0.3
Spectral gain (ms/mmHg)	3.03 (1.18-6.16)	0.59 (0.33-1.42)	0.01
Spectral coherence (0-1)	0.81 ± 0.02	0.93 ± 0.01	0.001
% time with coherence < 0.5	33.33 (20.64- 42.60)	0.00 (0.00-7.07)	0.006

All results were reported as means ± SEM when normality has been checked, as median (25-75 percentile) values otherwise. For each patient, the resulting spectral components values (except the last line, % time with coherence < 0.5) were the mean of the successive 16-second epoch values, when the spectral coherence between RR and SBP was greater than 0.5, allowing the gain analysis. The gain value was lower in the stable group, due to the decrease in HF-RR spectral density, whereas the spectral coherence was greater and the percentage of time with coherence < 0.5 was lower.

The spectral gain was lower in the stable group, because HF-RR spectral density was lower, whereas HF-SBP spectral density was not different. The percentage of time, in number of 16-second epochs, with a coherence greater than 0.5 between the RR and SBP spectra (allowing the gain calculation), and the spectral coherence itself were higher for the stable group.

At PEEP 10, the stable group had lower mean RR interval (Table 3) and mean arterial pressure (47 ± 4 versus 71 ± 6 mmHg, p = 0.003) than the unstable group.

**Table 3:** Comparison of the RR HF-CDM parameters between the two groups of patients (unstable *vs *stable) at PEEP 10.

RR series	Parameters	Unstable group (n = 11)	Stable group (n = 12)	*p *value
Raw RR	mean value (ms)	777 (613-911)	551 (473-600)	0.004
HF-CDM amplitude	mean value (ms)	3.04 (1.91-7.17)	1.33 (1.10-1.98)	0.007
	lnHF/RR (ms/s)	1.42 (0.80-2.18)	0.54 (0.15-1.28)	0.05
	SD (ms)	1.21 (0.81-2.03)	0.14 (0.09-0.30)	0.001
	time below threshold^a ^(%)	8.37 (4.50-11.92)	0.00 (0.00-0.00)	0.001
HF-CDM phase	SD (rad)	0.80 (0.35-2.42)	0.13 (0.08-0.22)	0.001
	maximal drift (rad)	2.76 (0.78-6.17)	0.44 (0.29-0.679)	0.001

All results were reported as means ± SEM when normality has been checked, as median (25-75 percentile) values otherwise. ^a^: percentage of time spent with HF amplitude below 50% of the mean HF amplitude for the individual patient. For each patient, the resulting parameter values were the average of the beat-to-beat values over the analysis period. Mean RR and HF amplitude were lower in the stable group. The two parameters related to the variability of HF amplitude (standard-deviation and time spent below the threshold of amplitude) were lower in this group, reflecting a more stable HF amplitude over time. The two parameters related to the variability of the HF-RR/ventilation phase difference (standard-deviation and maximal drifts) were lower in the stable group, reflecting a more stable RR/ventilation phase difference over time.

#### RR variability: HF-RR amplitude and cardiorespiratory phase difference over time (Table 3)

Mean HF-RR amplitude was lower in the stable group, even if normalized by the RR interval, as previously suggested [33]. HF-RR amplitude was steadier over time in the stable group, as reflected by lower standard deviation and lower time below the 50% threshold of the mean value. Cardiorespiratory phase difference was also steadier in the stable group, with lower standard deviation and lower maximal drift (Figure 1).

### Effects of PEEP change in the whole population

#### Baroreflex sensitivity (Figure 2)

**Figure 2:**
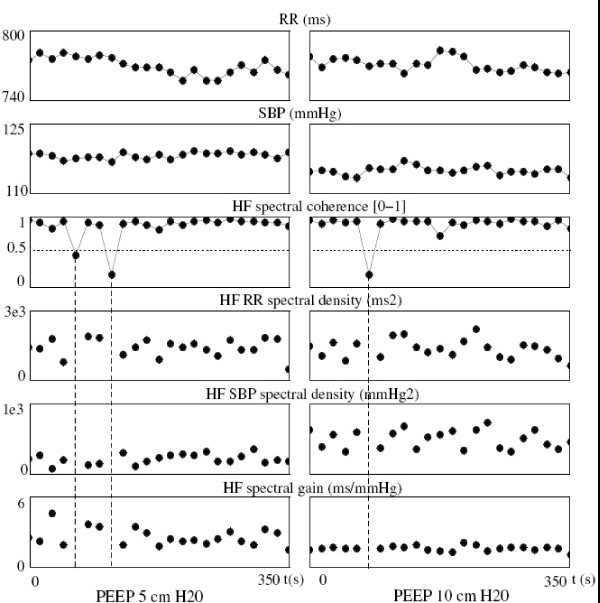
**Comparison of the spectral gain parameters between PEEP 5 and PEEP 10, in one subject, representative of the mean behavior of all patients**. Each dot represented an averaged value over 16 seconds. Raw RR and SBP remained at the same level, while the gain was lower at PEEP 10, due to the increase in SBP spectral density. The mean coherence value and the percentage of time (in number of 16-second epochs) with coherence under 0.5 did not significantly differ between PEEP 5 and PEEP 10. Dashed lines showed 3 epochs with low coherence, discarding them from the analysis.

The baroreflex gain was lower at PEEP 10 than at PEEP 5 (1.13 (0.51-3.10) vs 1.94 (0.64-3.86) ms/mmHg; p = 0.005). It was due to the increase in HF-SBP spectral density (1120 (576-3752) vs 771 (421-2975) mmHg^2^; p = 0.007) whereas HF-RR spectral density remained unchanged (1997 (675-10367) vs 2046 (816-8726) ms^2^; p = 0.5). The percentage of time, in the number of 16-second epochs, with a coherence between SBP and RR spectra lower than 0.5 (precluding the calculation of baroreflex gain) was significantly lower at PEEP 10 than at PEEP 5 (18.01 ± 4.04% vs 26.96 ± 4.71%; p = 0.04). Neither mean raw RR (650 (498-758) vs 592 (504-775) ms; p = 0.5), nor mean raw SBP (114 (102-131) vs 115 (102-130) mmHg; p = 0.2) differed from PEEP 5 to PEEP 10.

#### RR variability: HF-RR amplitude and cardiorespiratory phase difference over time

**Figure 3:**
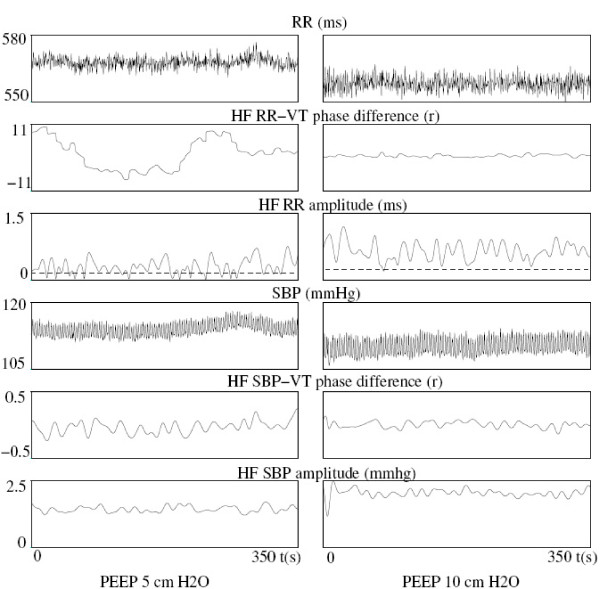
**Comparison of the CDM parameters between PEEP 5 and PEEP 10 in a subject representative of the mean behavior of all patients**. This subject exhibited great differences as he was classified in the unstable group at PEEP 5 and reclassified in the stable group at PEEP 10. So, the figure mainly focused on the greater variability of the HF phase and amplitude of RR and SBP, at PEEP 5. This greater variability was estimated by the standard-deviation of the time series, the maximal drift of the phase, the percentage of time spent below the amplitude threshold (dashed line).

Mean HF-RR amplitude did not differ between PEEP 5 and PEEP 10 (2.19 (1.20-3.43) vs 1.94 (1.20-3.50) ms; p = 0.5), that is in agreement with HF RR spectral density results. Nevertheless, time-variations of HF-RR amplitude and cardiorespiratory phase difference strongly differed between PEEP 5 and PEEP 10 (Figure 3). The lower percentage of time spent below the threshold (5.2 (0.0-16.4) % at PEEP 5 vs 0.0 (0.0-7.8) % at PEEP 10; p = 0.02) reflected a greater stability of HF-RR amplitude at PEEP 10. The cardiorespiratory phase difference was also more stable over time at PEEP 10 than at PEEP 5, as reflected by a lower standard-deviation (0.26 (0.13-0.77) vs 0.43 (0.14-0.75) rad; p = 0.05) and a lower maximal drift (0.68 (0.42-2.66) vs 1.53 (0.73-3.44) rad; p = 0.007).

At PEEP 5, 15 patients were in the unstable group and 8 in the stable group. At PEEP 10, 11 patients were in the unstable group (all of them were already in this group at PEEP 5) and 12 in the stable group. Thus, the general stabilizing effect of increasing PEEP on HF RR variability was especially pronounced in four patients and reflected by their transfer from unstable to stable group. Figure 3 represented one of those patients, who exhibited highly variable cardiorespiratory phase difference and HF-RR amplitude at PEEP 5, those parameters becoming more stable at PEEP 10, and even very stable for the phase difference.

## Discussion

### Main findings

In a previous study [15], we have observed that some mechanically ventilated patients had variable HF amplitude of RR variability and cardiorespiratory phase difference over time, and others did not. We hypothesized that a stable pattern could reflect a blunted autonomic nervous function. The present study has shown that patients with stable HF-RR amplitude and cardiorespiratory phase difference had lower baroreflex gain and lower mean HF-RR amplitude, suggesting an altered autonomic nervous function. Moreover, as PEEP has been shown to affect the autonomic nervous system in other settings, we also hypothesized that increasing PEEP could worsen the autonomic nervous function in mechanically ventilated patients. The present study has confirmed that increasing PEEP may decrease the baroreflex sensitivity and promote the stability of HF-RR amplitude and cardiorespiratory phase difference over time, two conditions associated with a blunted autonomic nervous function, as demonstrated above.

### Autonomic nervous system in critical care patients

HF RR variability and baroreflex gain have been frequently used to assess the autonomic nervous system in critical care patients [34,35], although the value of cardiovascular variability as an index of autonomic control of circulation remains controversial [36]. In critical care patients, a reduced RR variability has been already demonstrated in septic shock [1], trauma [2], acute brain injury [37] or multiple organ failure [3,34], and has been often interpreted as an autonomic dysfunction. But our study is the first to investigate the temporal changes of RR variability, in amplitude as well as in phase, and to show that monotonous phase and amplitude of RR variability might be associated with altered autonomic function. Thus, the temporal assessment of RR variability could provide a non-invasive index of autonomic nervous function. That is clinically relevant, because a real-time monitoring of RR variability is now available [38] and because autonomic dysfunction has been shown to increase the mortality of multiorgan failure patients [34]. Nevertheless, this real-time monitoring is not yet usually employed, and its feasibility remains to be demonstrated, because there are potential limitations, like signal artefacts or extrasystolic beats.

In our study, autonomic dysfunction occurred in approximately half of critically ill, mechanically ventilated patients. Few authors reported the prevalence of autonomic disorders in critical care patients, because of the lack of clear diagnostic criteria. Nevertheless, our results are in accordance with a study on 1425 trauma patients, which reported that 56% of the patients exhibited autonomic dysfunction [2]. Our population was relatively heterogeneous in terms of age, gender or comorbidities and this might have affect the occurrence of autonomic dysfunction, but we believe that such a high prevalence was most likely due to the acute illness. Thirteen of the 23 patients suffered from pneumonia. To our knowledge, there is no report of a higher prevalence of autonomic dysfunction in pneumonia, but most of these patients also had signs of sepsis and/or septic shock, two conditions well known to be associated with autonomic disorders [1].

### Effects of mechanical ventilation on the autonomic nervous system

During spontaneous breathing, many ventilatory parameters may affect the autonomic nervous system. For example, HF-RR amplitude increases when tidal volume increases or when breathing frequency decreases [39]. PaCO2 [40] or inspiratory-to-expiratory ratio [41] may also affect RR variability. In our study, ventilator settings were therefore standardized (except for the PEEP level) to ensure comparable influence on autonomic nervous function. We also checked that PaCO2 was not different between the two groups.

By contrast, the effect of mechanical, positive pressure ventilation on the autonomic nervous system was scarcely explored, especially in critical care patients. In two studies of low-risk patients under general anaesthesia, reversal of the phase of respiratory sinus arrhythmia was noted in 26 of 28 [42] and 3 of 10 patients [43], respectively. In critical care patients, our study previously reported extremely variable phases of respiratory sinus arrhythmia [15].

Focusing on the effect of PEEP, modulation of autonomic nervous function, and especially of the baroreflex gain, has been already described, but with conflicting results and never in critical care patients. In healthy subjects, several authors [44,45] observed an enhancement of the HF index of the spectral baroreflex gain at a PEEP of 5 mbar, compared to control subjects breathing without positive pressure. On the other hand, Valipour [13], using the sequence method, described a decline in the mean slope of spontaneous baroreceptor sequences at pressure levels > 10 cmH2O, compared to lower pressure levels (0, 3 or 5 cmH2O). Significant increases in the baroreflex gain were also described with PEEP in severe obstructive sleep apnea, with [46] or without [12] heart failure, and in snorers [47]. In critical care, mechanically ventilated patients, we observed a decrease in baroreflex sensitivity with increasing PEEP. These discrepancies could be explained by very different populations. Indeed, mean values of the baroreflex gain in the present study were lower than those of the above studies.

### Autonomic dysfunction: pathophysiological aspects

Healthy physiologic systems exhibit marked signal variability, while diseased systems show a loss of variability [37]. This "decomplexification" process has been linked to the severity and outcome of critical illness [37], and we suggest that excessively stable pattern of HF-RR amplitude and cardiorespiratory phase difference could reflect a decomplexification of cardiorespiratory interactions in our patients. This increased regularity could result from an uncoupling process. Indeed, it has been suggested that healthy organs behave as biological oscillators which couple to one another through a communications network including neural, humoral and cytokines components [48]. Diseased states would be associated with an uncoupling of these oscillators and an increased regularity of each oscillator (for example, an increased RR regularity). In critical care patients, uncoupling has been involved in multiple organ dysfunction syndrome [48], acute brain injury [49] or septic shock [50].

Several authors have emphasized the interactions between the central nervous system (including autonomic nervous centers) and other parts of the communications network mentioned above. Tight relations exist between immune and central nervous system [51]: during an injury or infection, brainstem centers receive sensory inputs from the immune system through humoral (circulating cytokines) and neural (afferent vagus nerve) routes, and may adjust the immune response through neuro-endocrine pathways or hard wired connections. Indeed, the stimulation of the hypothalamus-pituitary-adrenal axis exerts strong anti-inflammatory effects, as well as efferent vagal activation (release of acetylcholine by postganglionic neurons inhibits the release of pro-inflammatory cytokines by immune cells) [51]. Interactions between autonomic nervous system and endothelial function have been also described [52], involving oxidative stress, nitric oxide, insulin resistance or platelet activation. In critical care patients, most parts of this complex network are disturbed and could contribute to the autonomic dysfunction.

Many other factors present in critical care patients may interfere with the autonomic nervous system. Among sedative drugs, propofol has been shown to decrease the slope of baroreflex [7], whereas benzodiazepines decreased the high-frequency RR variability [53]. In septic shock, baroreflex gain and low-frequency/high-frequency ratio of RR variability were correlated with plasma norepinephrine levels [1]. These findings suggest a direct effect of administered vasoactive drugs on autonomic nervous system, although the administration of epinephrine or norepinephrine in healthy subjects has failed to demonstrate a change in baroreflex gain or RR variability [54]. Lastly, oxygenation status might also be involved, as hypoxemia has been shown to decrease the baroreflex gain in healthy volunteers [55] and the RR variability in chronic obstructive pulmonary disease [56]. But hypoxemia is unlikely to explain our results. Indeed, our patients, although suffering from acute lung injury, were not hypoxemic because FiO2 was adjusted to ensure safe PaO2 values.

### Differentiation between the two groups

We believe that an independent autonomic function likely of central origin, not coordinated with the mechanical ventilation, could still exist in some critical care, mechanically ventilated patients (unstable group) and not in others (stable group). This could account for the sudden shifts of HF-RR phase and amplitude observed in the unstable group. Interestingly, we have observed significantly lower RR interval and mean arterial pressure at PEEP 10 in patients belonging to the stable group, compared with those from the unstable group. These results might suggest that patients of the stable group were more hypovolemic and thus more affected by increasing PEEP. Indeed, in this group, PEEP 10 induced a decrease in arterial pressure (through a plausible reduced left ventricular stroke volume [57]) and a compensatory decrease in RR intervals, whereas no RR or arterial pressure variation was observed in the unstable group. Decreased RR intervals and arterial pressure were well described in hypovolemic humans [58], and PEEP-induced hemodynamic changes have been proposed as indicators of hypovolemia [57]. Nevertheless, the hypothesis that stable cardiorespiratory phase difference and HF-RR amplitude, which characterized the stable group, could be associated with hypovolemic states would need further confirmation. Indeed, another explanation for the shorter RR interval in the stable group at PEEP 10 could be the loss of autonomic (vagal) function.

Interestingly, 4 patients had an unstable pattern of variability at PEEP 5, which became stable at PEEP 10. To our knowledge, this observation that some mechanically ventilated patients might have independent autonomic function at PEEP 5 and will lose it at PEEP 10 has never been described, and is clinically relevant because of the prognostic value of the autonomic dysfunction [34]. PEEP is widely used and recommended [14] in the management of critical care patients with acute lung injury, in order to reduce the proportion of nonaerated lung and to improve arterial oxygenation. Deleterious effects of PEEP are well known, like circulatory depression [59], increased pulmonary edema [60] and overdistension [21], but its effect on autonomic nervous system has been poorly explored in critical care patients. An animal study on acute brain damaged rabbits has described a depressed autonomic nervous activity induced by PEEP [61], but our study is the first to present similar results in patients.

### Limitations of the study

A limitation of our study was the lack of data related to the response to PEEP in terms of respiratory mechanics and gas exchange. Indeed, data on respiratory mechanics would have allowed checking the increased mechanical constraint induced at PEEP 10. Gas exchange measurements would have allowed investigating other factors known to affect RR variability, like PaCO2 or chemoreflex. These parameters were not recorded in the present study, because our objective was only to assess whether increasing PEEP could affect RR variability and baroreflex, and not to investigate the mechanisms responsible for this effect, whatever they were (direct mechanical effect, change in PaCO2 or PaO2, etc...). Therefore, we believe that the lack of respiratory mechanics and gas exchange does not question the reliability of our results.

Another limitation was that we failed to demonstrate an impact of the steady pattern of HF-RR amplitude and cardiorespiratory phase difference on mortality. The stable group had significantly higher SOFA score and vasoactive drugs dosage compared with the unstable one, strongly suggesting an increased severity. Although the mortality rates did not differ between the two groups, the study was not designed to assess the prognosis. A larger study would be interesting in order to confirm an impact on mortality.

## Conclusions

In critical care, mechanically ventilated patients, time-variations of HF-RR amplitude and cardiorespiratory phase difference could reflect the autonomic nervous system function. A steady pattern was associated with decreased baroreflex sensitivity and RR variability, suggesting impaired autonomic nervous system. Increasing PEEP reduced the baroreflex sensitivity, promoted a steady pattern, and could therefore alter the autonomic nervous function in these patients.

## List Of Abbreviations Used

RR: intervals between consecutive R waves on the electrocardiogram; PEEP: positive end-expiratory pressure; HF: high-frequency; ECG: electrocardiogram; SBP: systolic blood pressure; ICU: intensive care unit; ALI: acute lung injury; Vt: tidal volume; FiO2: inspired oxygen fraction; SAPS: simplified acute physiology score; SOFA: sequential organ failure assessment score; PaO2: oxygen partial pressure in arterial blood; PCO2: carbon dioxide partial pressure in arterial blood; BP: blood pressure; CDM: complex demodulation; RSA: respiratory sinus arrhythmia; BRS: baroreflex sensitivity

## Competing interests

The authors declare that they have no competing interests.

## Authors' contributions

AVDL conceived the study, acquired the data of patients and drafted part of the manuscript. CM carried out the signal analysis, performed the statistical analysis and drafted part of the manuscript. YP and FC participated in the design of the study and in the interpretation of the results. FC was responsible for study coordination. All authors read and approved the final manuscript.

## References

[B1] Annane D Trabold F Sharshar T Jarrin I Blanc AS Raphael JC Gajdos P Inappropriate sympathetic activation at onset of septic shock; a spectral analysis approach Am J Respir Crit Care Med 1999 160 458 465 10430714 10.1164/ajrccm.160.2.9810073

[B2] Morris JA Norris PR Ozdas A Waitman LR Harrell FE Jr Williams AE Cao H Jenkins JM Reduced heart rate variability: an indicator of cardiac uncoupling and diminished physiologic reserve in 1425 trauma patients J Trauma 2006 60 1165 1174 10.1097/01.ta.0000220384.04978.3b 16766957

[B3] Papaioannou VE Maglaveras N Houvarda I Antoniadou E Vretzakis G Investigation of altered heart rate variability, nonlinear properties of heart rate signals, and organ dysfunction longitudinally over time in intensive care unit patients J Crit Care 2006 21 1 95 103 10.1016/j.jcrc.2005.12.007 16616632

[B4] La Rovere MT Pinna GD Raczak G Baroreflex sensitivity: measurement and clinical implications Ann Noninvasive Electrocardiol 2008 13 2 191 207 10.1111/j.1542-474X.2008.00219.x 18426445 PMC6931942

[B5] Manzella D Paolisso G Cardiac autonomic activity and Type II diabetes mellitus Clin Sci (Lond) 2005 108 2 93 99 10.1042/CS20040223 15476437

[B6] La Rovere MT Pinna GD Hohnloser SH Marcus FI Mortara A Nohara R Bigger JT Jr Camm AJ Schwartz PJ ATRAMI Investigators Baroreflex sensitivity and heart rate variability in the identification of patients at risk for life-threatening arrhythmias: implications for clinical trials Circulation 2001 103 16 2072 2077 11319197 10.1161/01.cir.103.16.2072

[B7] Ebert TJ Sympathetic and hemodynamic effects of moderate and deep sedation with propofol in humans Anesthesiology 2005 103 1 20 24 10.1097/00000542-200507000-00007 15983452

[B8] Jordan J Tank J Shannon JR Diedrich A Lipp A Schröder C Arnold G Sharma AM Biaggioni I Robertson D Luft FC Baroreflex buffering and susceptibility to vasoactive drugs Circulation 2002 105 12 1459 1464 10.1161/01.CIR.0000012126.56352.FD 11914255

[B9] Butler GC Naughton MT Rahman MA Bradley TD Floras JS Continuous positive airway pressure increases heart rate variability in congestive heart failure J Am Coll Cardiol 1995 25 3 672 679 10.1016/0735-1097(94)00427-R 7860912

[B10] Gilman MP Floras JS Usui K Kaneko Y Leung RS Bradley TD Continuous positive airway pressure increases heart rate variability in heart failure patients with obstructive sleep apnoea Clin Sci (Lond) 2008 114 3 243 249 10.1042/CS20070172 17824846

[B11] Khoo MC Belozeroff V Berry RB Sassoon CS Cardiac autonomic control in obstructive sleep apnea: effects of long-term CPAP therapy Am J Respir Crit Care Med 2001 164 5 807 812 11549537 10.1164/ajrccm.164.5.2010124

[B12] Bonsignore MR Parati G Insalaco G Castiglioni P Marrone O Romano S Salvaggio A Mancia G Bonsignore G Di Rienzo M Baroreflex control of heart rate during sleep in severe obstructive sleep apnoea: effects of acute CPAP Eur Respir J 2006 27 1 128 135 10.1183/09031936.06.00042904 16387945

[B13] Valipour A Schneider F Kössler W Saliba S Burghuber OC Heart rate variability and spontaneous baroreflex sequences in supine healthy volunteers subjected to nasal positive airway pressure J Appl Physiol 2005 99 6 2137 2143 10.1152/japplphysiol.00003.2005 16002778

[B14] Brower RG Lanken PN MacIntyre N Matthay MA Morris A Ancukiewicz M Schoenfeld D Thompson BT National Heart, Lung, and Blood Institute ARDS Clinical Trials Network Higher versus lower positive end-expiratory pressures in patients with the acute respiratory distress syndrome N Engl J Med 2004 351 4 327 336 10.1056/NEJMoa032193 15269312

[B15] Van de Louw A Médigue C Papelier Y Cottin F Breathing cardiovascular variability and baroreflex in mechanically ventilated patients Am J Physiol Regul Integr Comp Physiol 2008 295 6 R1934 1940 18922962 10.1152/ajpregu.90475.2008

[B16] Porta A Gnecchi-Ruscone T Tobaldini E Guzzetti S Furlan R Montano N Progressive decrease of heart period variability entropy-based complexity during graded head-up tilt J Appl Physiol 2007 103 4 1143 1149 10.1152/japplphysiol.00293.2007 17569773

[B17] Seely AJ Macklem PT Complex systems and the technology of variability analysis Crit Care 2004 8 6 R367 384 10.1186/cc2948 15566580 PMC1065053

[B18] Bernard GR Artigas A Brigham KL Carlet J Falke K Hudson L Lamy M Legall JR Morris A Spragg R The American-European Consensus Conference on ARDS. Definitions, mechanisms, relevant outcomes, and clinical trial coordination Am J Respir Crit Care Med 1994 149 818 824 7509706 10.1164/ajrccm.149.3.7509706

[B19] Le Gall JR Lemeshow S Saulnier F A new Simplified Acute Physiology Score (SAPS II) based on a European/North American multicenter study JAMA 1993 270 24 2957 2963 10.1001/jama.270.24.2957 8254858

[B20] Vincent JL de Mendonça A Cantraine F Moreno R Takala J Suter PM Sprung CL Colardyn F Blecher S Use of the SOFA score to assess the incidence of organ dysfunction/failure in intensive care units: results of a multicenter, prospective study. Working group on "sepsis-related problems" of the European Society of Intensive Care Medicine Crit Care Med 1998 26 11 1793 1800 9824069 10.1097/00003246-199811000-00016

[B21] Villar J Herrera-Abreu MT Valladares F Muros M Pérez-Méndez L Flores C Kacmarek RM Experimental ventilator-induced lung injury: exacerbation by positive end-expiratory pressure Anesthesiology 2009 110 6 1341 1347 10.1097/ALN.0b013e31819fcba9 19417614

[B22] Zhang Q Illanes Manriquez A Médigue C Papelier Y Sorine M An algorithm for robust and efficient location of T-wave ends in electrocardiograms IEEE Trans Biomed Eng 2006 53 12 Pt 1 2544 2552 10.1109/TBME.2006.884644 17153212

[B23] Mateika JH Omran Q Rowley JA Zhou XS Diamond MP Badr MS Treatment with leuprolide acetate decreases the threshold of the ventilatory response to carbon dioxide in healthy males J Physiol 2004 561 Pt 2 637 646 10.1113/jphysiol.2004.071811 15375194 PMC1665369

[B24] Cottin F Médigue C Leprêtre PM Papelier Y Koralsztein JP Billat V Heart rate variability during exercise performed below and above ventilatory threshold Med Sci Sports Exerc 2004 36 4 594 600 10.1249/01.MSS.0000121982.14718.2A 15064586

[B25] Hayano J Taylor JA Mukai S Okada A Watanabe Y Takata K Fujinami T Assessment of frequency shifts in R-R interval variability and respiration with complex demodulation J Appl Physiol 1994 77 6 2879 2888 7896636 10.1152/jappl.1994.77.6.2879

[B26] Mangin L Monti A Médigue C Cardiorespiratory system dynamics in chronic heart failure European Journal of Heart Failure 2002 5 617 625 10.1016/S1388-9842(02)00098-3 12413506

[B27] Monti A Médigue C Mangin L Instantaneous parameter estimation in cardiovascular time series by harmonic and time-frequency analysis IEEE trans Biomed Eng 2002 49 12 1547 1556 10.1109/TBME.2002.805478 12549736

[B28] Task force of the European Society of Cardiology and the North American Society of Pacing and Electrophysiology Heart rate variability: Standards of measurement, Physiological Interpretation, and clinical use Circulation 1996 93 1043 1065 8598068

[B29] De Boer RW Karemaker JM Strackee J Relationships between short-term blood-pressure fluctuations and heart-rate variability in resting subjects. I: A spectral analysis approach Med Biol Eng Comput 1985 23 4 352 358 10.1007/BF02441589 4046655

[B30] Taylor JA Eckberg DL Fundamental relations between short-term RR interval and arterial blood pressure oscillations in humans Circulation 1996 93 1527 1532 8608621 10.1161/01.cir.93.8.1527

[B31] Maestri R Pinna GD Mortara A La Rovere MT Tavazzi L Assessing baroreflex sensitivity in post-myocardial infarction patients; comparison of spectral and phenylephrine techniques J Am Coll Cardiol 1998 31 344 351 10.1016/S0735-1097(97)00499-3 9462578

[B32] Mangin L Monti A Médigue C Macquin-Mavier I Lopes M Gueret P Castaigne A Swynghedauw B Mansier P Altered baroreflex gain during voluntary breathing in chronic heart failure Eur J Heart Fail 2001 3 2 189 195 10.1016/S1388-9842(00)00147-1 11246056

[B33] Kiviniemi AM Hautala AJ Seppänen T Mäkikallio TH Huikuri HV Tulppo MP Saturation of high-frequency oscillations of R-R intervals in healthy subjects and patients after acute myocardial infarction during ambulatory conditions Am J Physiol Heart Circ Physiol 2004 287 5 H1921 1927 10.1152/ajpheart.00433.2004 15242837

[B34] Schmidt H Müller-Werdan U Hoffmann T Francis DP Piepoli MF Rauchhaus M Prondzinsky R Loppnow H Buerke M Hoyer D Werdan K Autonomic dysfunction predicts mortality in patients with multiple organ dysfunction syndrome of different age groups Crit Care Med 2005 33 9 1994 2002 10.1097/01.CCM.0000178181.91250.99 16148471

[B35] Fathizadeh P Shoemaker WC Wo CC Colombo J Autonomic activity in trauma patients based on variability of heart rate and respiratory rate Crit Care Med 2004 32 6 1300 1305 10.1097/01.CCM.0000127776.78490.E4 15187510

[B36] Parati G Mancia G Di Rienzo M Castiglioni P Taylor JA Studinger P Point: Counterpoint: cardiovascular variability is/is not an index of autonomic control of circulation J Appl Physiol 2006 101 2 676 682 10.1152/japplphysiol.00446.2006 16645191

[B37] Goldstein B Fiser DH Kelly MM Mickelsen D Ruttimann U Pollack MM Decomplexification in critical illness and injury: relationship between heart rate variability, severity of illness, and outcome Crit Care Med 1998 26 2 352 357 10.1097/00003246-199802000-00040 9468175

[B38] Kasaoka S Nakahara T Kawamura Y Tsuruta R Maekawa T Real-time monitoring of heart rate variability in critically ill patients J Crit Care 2009 in press 19781905 10.1016/j.jcrc.2009.06.047

[B39] Hirsch JA Bishop B Respiratory sinus arrhythmia in humans: how breathing pattern modulates heart rate Am J Physiol 1981 241 4 H620 629 7315987 10.1152/ajpheart.1981.241.4.H620

[B40] Pöyhönen M Syväoja S Hartikainen J Ruokonen E Takala J The effect of carbon dioxide, respiratory rate and tidal volume on human heart rate variability Acta Anaesthesiol Scand 2004 48 1 93 101 10.1111/j.1399-6576.2004.00272.x 14674979

[B41] Strauss-Blasche G Moser M Voica M McLeod DR Klammer N Marktl W Relative timing of inspiration and expiration affects respiratory sinus arrhythmia Clin Exp Pharmacol Physiol 2000 27 8 601 606 10.1046/j.1440-1681.2000.03306.x 10901389

[B42] Yli-Hankala A Porkkala T Kaukinen S Häkkinen V Jäntti V Respiratory sinus arrhythmia is reversed during positive pressure ventilation Acta Physiol Scand 1991 141 3 399 407 10.1111/j.1748-1716.1991.tb09097.x 1858511

[B43] Larsen PD Trent EL Galletly DC Cardioventilatory coupling: effects of IPPV Br J Anaesth 1999 82 4 546 550 10472220 10.1093/bja/82.4.546

[B44] Fietze I Romberg D Glos M Endres S Theres H Witt C Somers VK Effects of positive-pressure ventilation on the spontaneous baroreflex in healthy subjects J Appl Physiol 2004 96 3 1155 1160 10.1152/japplphysiol.00578.2003 14607849

[B45] Garet M Barthélémy JC Degache F Pichot V Duverney D Roche F Modulations of human autonomic function induced by positive pressure-assisted breathing Clin Physiol Funct Imaging 2006 26 1 15 20 10.1111/j.1475-097X.2005.00645.x 16398665

[B46] Ruttanaumpawan P Gilman MP Usui K Floras JS Bradley TD Sustained effect of continuous positive airway pressure on baroreflex sensitivity in congestive heart failure patients with obstructive sleep apnea J Hypertens 2008 26 6 1163 8 10.1097/HJH.0b013e3282fb81ed 18475154

[B47] Gates GJ Mateika SE Basner RC Mateika JH Baroreflex sensitivity in nonapneic snorers and control subjects before and after nasal continuous positive airway pressure Chest 2004 126 3 801 807 10.1378/chest.126.3.801 15364759

[B48] Godin PJ Buchman TG Uncoupling of biological oscillators: a complementary hypothesis concerning the pathogenesis of multiple organ dysfunction syndrome Crit Care Med 1996 24 7 1107 1116 10.1097/00003246-199607000-00008 8674321

[B49] Goldstein B Toweill D Lai S Sonnenthal K Kimberly B Uncoupling of the autonomic and cardiovascular systems in acute brain injury Am J Physiol 1998 275 4 Pt 2 R1287 1292 9756562 10.1152/ajpregu.1998.275.4.R1287

[B50] Ellenby MS McNames J Lai S McDonald BA Krieger D Sclabassi RJ Goldstein B Uncoupling and recoupling of autonomic regulation of the heart beat in pediatric septic shock Shock 2001 16 4 274 277 10.1097/00024382-200116040-00007 11580109

[B51] van Westerloo DJ Giebelen IAJ Poll T van der Vincent JL The Central and Autonomic Nervous Systems: Essential Regulators of the Immune Response Yearbook of Intensive Care and Emergency Medicine 2005 Springer Berlin Heidelberg 421 433 full_text

[B52] Harris KF Matthews KA Interactions between autonomic nervous system activity and endothelial function: a model for the development of cardiovascular disease Psychosom Med 2004 66 2 153 164 10.1097/01.psy.0000116719.95524.e2 15039499

[B53] Unoki T Grap MJ Sessler CN Best AM Wetzel P Hamilton A Mellott KG Munro CL Autonomic nervous system function and depth of sedation in adults receiving mechanical ventilation Am J Crit Care 2009 18 1 42 50 10.4037/ajcc2009509 19116404 PMC3690647

[B54] Tulen JH Man in 't Veld AJ Van Roon AM Moleman P Van Steenis HG Blankestijn PJ Boomsma F Spectral analysis of hemodynamics during infusions of epinephrine and norepinephrine in men J Appl Physiol 1994 76 5 1914 1921 8063650 10.1152/jappl.1994.76.5.1914

[B55] Steinback CD Salzer D Medeiros PJ Kowalchuk J Shoemaker JK Hypercapnic vs. hypoxic control of cardiovascular, cardiovagal, and sympathetic function Am J Physiol Regul Integr Comp Physiol 2009 296 2 R402 410 19091913 10.1152/ajpregu.90772.2008

[B56] Chen WL Chen GY Kuo CD Hypoxemia and autonomic nervous dysfunction in patients with chronic obstructive pulmonary disease Respir Med 2006 100 9 1547 1553 10.1016/j.rmed.2006.01.006 16488587

[B57] Lambert P Sloth E Smith B Hansen LK Koefoed-Nielsen J Tønnesen E Larsson A Does a positive end-expiratory pressure-induced reduction in stroke volume indicate preload responsiveness? An experimental study Acta Anaesthesiol Scand 2007 51 4 415 425 10.1111/j.1399-6576.2007.01248.x 17378779

[B58] Skillman JJ Hedley-Whyte J Pallotta JA Cardiorespiratory, metabolic and endocrine changes after hemorrhage in man Ann Surg 1971 174 6 911 922 10.1097/00000658-197112000-00006 5132436 PMC1397657

[B59] Pinsky MR The hemodynamic consequences of mechanical ventilation: an evolving story Intensive Care Med 1997 23 5 493 503 10.1007/s001340050364 9201520

[B60] Toung TJ Saharia P Mitzner WA Permutt S Cameron JL The beneficial and harmful effects of positive end expiratory pressure Surg Gynecol Obstet 1978 147 4 518 524 360447

[B61] Kurita S Kawamoto M Hidaka S Yuge O Positive end-expiratory pressure depressed cardiovascular autonomic nervous system activity in acute brain damaged rabbits under general anesthesia Hiroshima J Med Sci 2003 52 4 59 67 14760994

